# Sports activity and quality of life improve after isolated ACL, isolated PCL, and combined ACL/PCL reconstruction

**DOI:** 10.1007/s00167-022-07060-w

**Published:** 2022-07-09

**Authors:** Philipp W. Winkler, Bálint Zsidai, Eric Narup, Janina Kaarre, Alexandra Horvath, Mikael Sansone, Eleonor Svantesson, Eric Hamrin Senorski, Volker Musahl, Kristian Samuelsson

**Affiliations:** 1 Department for Orthopaedic Sports Medicine, Klinikum Rechts der Isar, Technical University of Munich, Ismaninger Straße 22, 81675 Munich, Germany; 2 Department of Orthopaedics, Institute of Clinical Sciences, Sahlgrenska Academy, University of Gothenburg, Gothenburg, Sweden; 3 Department of Internal Medicine and Clinical Nutrition, Institute of Medicine, Sahlgrenska Academy, University of Gothenburg, Gothenburg, Sweden; 4 Department of Orthopaedics, Sahlgrenska University Hospital, Mölndal, Sweden; 5 Department of Health and Rehabilitation, Institute of Neuroscience and Physiology, Sahlgrenska Academy, University of Gothenburg, Gothenburg, Sweden; 6 Department of Orthopaedic Surgery, UPMC Freddie Fu Sports Medicine Center, University of Pittsburgh, Pittsburgh, PA USA

**Keywords:** KOOS, Patient-reported outcomes, Multiligament knee injury, Schenck classification, ACL, Anterior cruciate ligament, Posterior cruciate ligament, PCL

## Abstract

**Purpose:**

To compare patient-reported outcomes following isolated anterior cruciate ligament reconstruction (ACL-R), isolated posterior cruciate ligament reconstruction (PCL-R), and combined ACL-R and PCL-R (ACL/PCL-R), at a minimum follow-up of 2 years.

**Methods:**

This was a prospective observational registry cohort study based on the Swedish National Knee Ligament Registry. Patients undergoing isolated ACL-R, isolated PCL-R, and combined ACL/PCL-R between 2005 and 2019 were eligible for inclusion. Demographic characteristics as well as injury- and surgery-related data were queried from the SNKLR. To evaluate functional outcomes, the Knee Injury and Osteoarthritis Outcome Score (KOOS) was collected preoperatively and at 1- and 2-year follow-ups and compared between the treatment groups.

**Results:**

In total, 45,169 patients underwent isolated ACL-R, 192 patients isolated PCL-R, and 203 patients combined ACL/PCL-R. Preoperatively, and at the 1- and 2-year follow-ups, KOOS subscales were highest for the isolated ACL-R group, followed by the isolated PCL-R, and lowest for the combined ACL/PCL-R groups. Significant improvements were observed across all treatment groups in the majority of KOOS subscales between the preoperative, and 1- and 2-year follow-ups. All treatment groups showed the greatest improvements between the preoperative and 2-year follow-ups in the knee-related quality of life (mean improvement: isolated ACL-R, + 28 points; isolated PCL-R, + 23 points; combined ACL/PCL-R, + 21 points) and the function in sport and recreation (mean improvement: isolated ACL-R, + 26 points; isolated PCL-R, + 20 points; combined ACL/PCL-R, + 19 points) subscales.

**Conclusion:**

Clinically relevant improvements in knee function can be expected after isolated ACL-R, isolated PCL-R, and combined ACL/PCL-R. Functional improvements were particularly pronounced in the KOOS function in sport and recreation subscale, indicating the importance of knee stability for sports activity. This study facilitates more comprehensive patient education about functional expectations after surgical treatment of isolated and combined ACL and PCL injuries.

**Level of evidence:**

Level 2.

## Introduction

The interaction of passive and dynamic anatomical components of the knee facilitates a wide range of motion in six degrees of freedom. An indispensable part of this system is the central pivot, consisting of the anterior cruciate ligament (ACL) and posterior cruciate ligament (PCL) [11]. Whilst the ACL and PCL together act as the primary restraint against anterior and posterior tibial translation, both have a salient contribution to rotatory stability of the knee, namely internal/external as well as varus/valgus tibial rotation [4, 11, 14]. Isolated ACL injuries are common, and account for up to 50% of sports-related knee injuries [6, 17]. In contrast, isolated PCL injuries are rare with a reported incidence of 1–6% [26]. However, PCL injuries most frequently occur as combined injuries, with concurrent ACL and PCL injuries (i.e. bicruciate injury or Schenck type KDII) having a reported prevalence of 4–7% amongst PCL-injured knees [5, 12, 16, 18, 24, 29].

Despite differences in physiologic function, demographic characteristics, and injury mechanisms, surprisingly, one study showed similar patient-reported outcomes (PROs) and patient satisfaction rates after isolated ACL reconstruction (ACL-R) and isolated PCL reconstruction (PCL-R) after a minimum 2-year follow-up [12]. Conversely, a recent investigation by the Norwegian National Knee Ligament Registry (NKLR) demonstrated lower preoperative PROs in patients with isolated PCL injury compared to patients with isolated ACL injury, indicating the severity of PCL tears [2]. With regard to bicruciate injuries, several case series with less than 35 patients collectively display significant improvement in clinical and functional outcomes after single-stage combined ACL and PCL reconstruction (ACL/PCL-R) [3, 7, 9, 15, 22, 28]. However, rates of return to sport after bicruciate reconstruction are low and vary considerably between studies, ranging from 19 to 85% [7, 15, 22, 28].

Given the controversies in outcomes between different injury patterns of the central pivot [8], there is a need for further investigation to guide clinicians in patient counselling. Consequently, the treatment strategy for patients with isolated and combined ACL and PCL injuries can be tailored more effectively to the individual needs of the patient. Therefore, the objective of this study was to compare Knee Injury and Osteoarthritis Outcome Score (KOOS) subscales between patients undergoing isolated ACL-R, isolated PCL-R, and combined ACL/PCL-R based on the Swedish National Knee Ligament Registry (SNKLR) with a minimum 2-year follow-up. It was hypothesised that isolated ACL-R would result in superior functional scores based on KOOS subscales compared to isolated PCL-R and combined ACL/PCL-R. Furthermore, it was hypothesised that isolated PCL-R would yield superior functional outcomes than combined ACL/PCL-R.

## Materials and methods

This study was approved by the Swedish Ethical Review Authority (Dnr 2020-03559 and 2021-01002) and was performed in accordance with the Declaration of Helsinki. This cohort study was based on the SNKLR. Participation in the SNKLR is voluntary for patients and surgeons, and no written informed consent is necessary for enrolment. The registry complies with the Swedish legislation relating to data security. The SNKLR represents a nationwide database established in 2005 with prospectively collected patient-, injury-, and surgery-related data on knee ligament injuries. All operating units and hospitals across Sweden participate in data collection with a coverage to the SNKLR regarding ACL-R of approximately > 90% [1].

Patients undergoing operative treatment of knee ligament injuries are included in the SNKLR. A complete data input into the registry consists of a surgeon-related part and a patient-related part. After a knee ligament reconstruction, the operating surgeon enters the required data into the register. Patient-related data comprise patient age, sex, and body mass index (BMI). Injury-related data include injury mechanism (sports related, traffic related, other), laterality of the affected knee, and concomitant meniscal, cartilage, neurovascular, and ligament injuries. Surgery-related data include time from injury to surgery, type of ACL-R/PCL-R (primary vs. revision), graft type, femoral and tibial graft fixation technique (suspensory, interference screw, others), and concurrent operative procedures. Preoperatively and at 1- and 2-year follow-ups, each patient is asked to complete a questionnaire consisting of the KOOS. The KOOS is a validated, self-administered outcome instrument consisting of 5 subscales and 42 questions to assess both short-term and long-term outcomes in patients with knee injury and posttraumatic osteoarthritis [23]. The KOOS has been validated for several different languages, including Swedish, and the highest effect size was reported for the knee-related quality of life subscale [23]. Both surgeon-related and patient-related parts are entered into the registry via a secure online portal or by paper questionnaires.

**Fig. 1:**
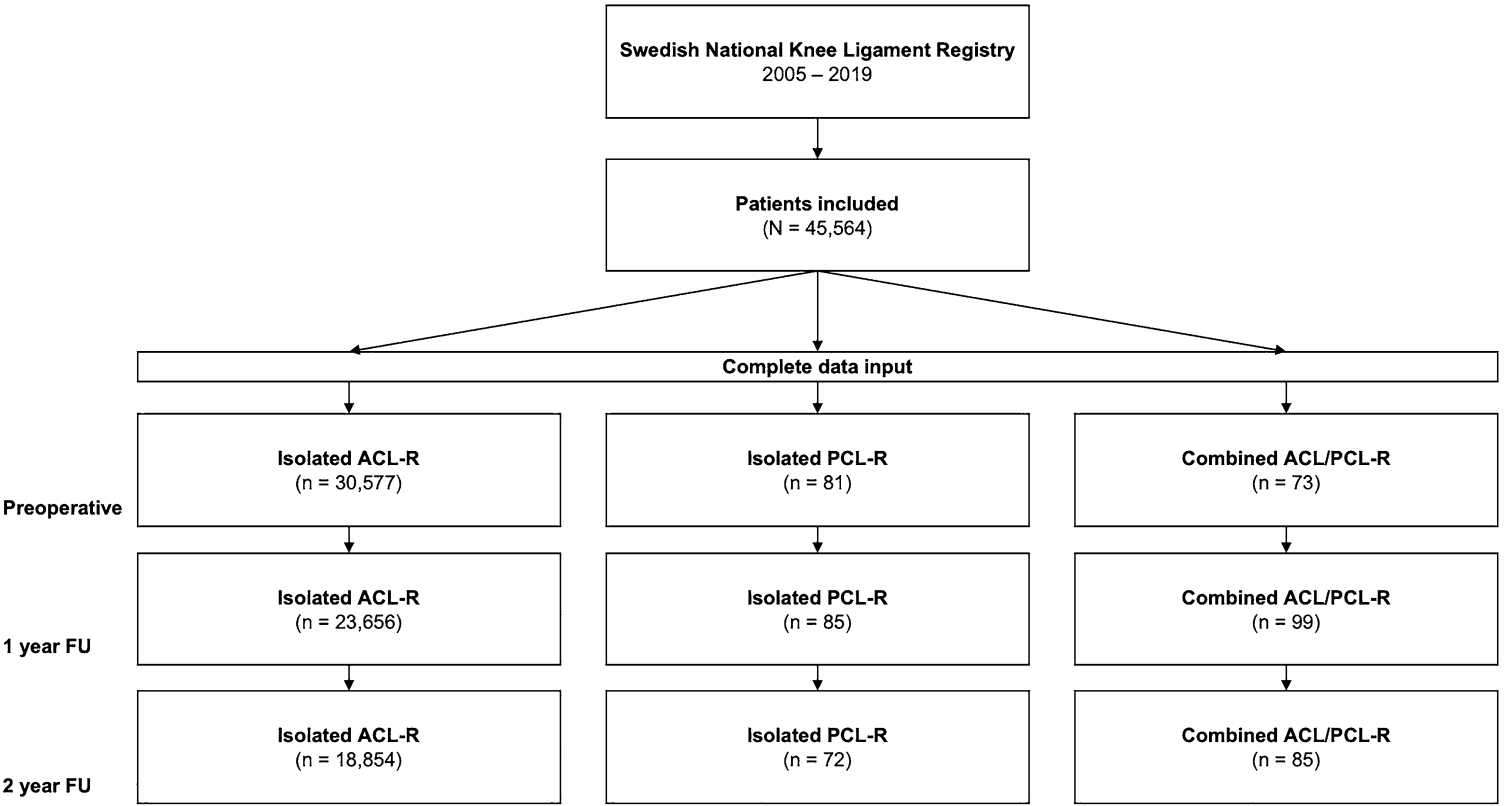
Flow-chart of patient enrollment. Complete data input refers to data available for all KOOS subscales. *ACL-R* anterior cruciate ligament reconstruction, *ACL/PCL-R* combined anterior cruciate ligament and posterior cruciate ligament reconstruction, *FU* follow-up, *PCL-R* posterior cruciate ligament reconstruction

After data extraction, patients were categorised according to the injury pattern of the central pivot as follows: isolated ACL-R, isolated PCL-R, and combined ACL/PCL-R (Fig. 1).

### Eligibility criteria

Patients undergoing primary isolated ACL-R, primary isolated PCL-R, and combined ACL/PCL-R (i.e. bicruciate) between January 1, 2005 and December 31, 2019 were included in this study. Patients with concomitant medial collateral ligament reconstruction, lateral collateral ligament reconstruction, posterolateral corner reconstruction, or lateral extra-articular tenodesis were excluded. Anterior cruciate ligament reconstruction and PCL-R were considered “isolated” if there was no concomitant ligament reconstruction recorded in the SNKLR. Patients with concomitant meniscal and/or cartilage injury/treatment were considered for inclusion.

### Statistical analysis

Statistical analysis was performed using SAS software version 9.4 (SAS Institute, Cary, NC USA). Continuous variables are expressed as mean and standard deviation (SD). Categorical variables are presented as count (*n*) and proportion (%). Levene’s test and normality checks were carried out. The Kruskal–Wallis test was used to compare preoperative KOOS subscales between groups (isolated ACL-R vs. isolated PCL-R vs. combined ACL/PCL-R). Pairwise comparisons of preoperative KOOS subscales and improvements in KOOS subscales over time were performed using Fisher’s non-parametric permutation test, respectively. Improvements in KOOS subscales over time within groups (preoperative vs. 1-year follow-up vs. 2-year follow-up) were compared using the Wilcoxon Signed Rank test. One-way analysis of covariance (ANCOVA) analyses were conducted to compare KOOS subscales between groups (isolated ACL-R vs. isolated PCL-R vs. combined ACL/PCL-R) at 1- and 2-year follow-ups whilst controlling for preoperative subscale values, followed by post hoc pairwise comparison using the Bonferroni method. The level of significance was set at *p* < 0.05.

## Results

Over the 15-year period analyzed in this study (2005–2019), 45,564 patients met the inclusion criteria. In total, 45,169 patients underwent isolated ACL-R, 192 patients underwent isolated PCL-R, and 203 patients underwent combined ACL/PCL-R. The flowchart depicted in Fig. 1 shows the number of patients with complete data input (i.e. data available for all KOOS subscales) at the 1- and 2-year follow-ups. Demographic characteristics and injury-related data of the included patients are shown in Table 1.

**Table 1:** Demographic characteristics and injury-related data

Variable	ACL-R (*n* = 45,169)	PCL-R (*n* = 192)	ACL/PCL-R (*n* = 203)	*p* value
Age, (years)	27 ± 10.4 (7–74)	30 ± 11.8 (11–61)	34 ± 12.9 (8–67)	< 0.001^a^
Males, *n* (%)	25,537 (57)	115 (60)	131 (65)	0.047^b^
BMI, (kg/m^2^)	25 ± 3.4 (15–47)	25 ± 3.4 (20–37)	27 ± 5.5 (19–44)	< 0.001^a^
Right knee, *n* (%)	23,530 (52)	82 (43)	95 (47)	0.011^b^
Injury to surgery, (months)^c^	8 (4–18)	18 (9–31)	7 (1–14)	< 0.001^d^
Injury mechanism				< 0.001^b^
Sports-related, *n* (%)	40,085 (89)	122 (64)	109 (54)	
Traffic-related, *n* (%)	817 (2)	38 (20)	54 (27)	
Other, *n* (%)	4182 (9)	32 (17)	40 (20)	
Concomitant injury, *n* (%)	25,132 (56)	85 (44)	120 (59)	0.004^b^
Meniscus injury, *n* (%)	20,190 (45)	31 (16)	62 (31)	< 0.001^b^
Medial meniscus injury, *n* (%)	12,136 (27)	17 (9)	37 (18)	< 0.001^b^
Lateral meniscus injury, *n* (%)	11,425 (25)	16 (8)	36 (18)	< 0.001^b^

Continuous variables are expressed as mean ± standard deviation (range). Categorical variables are expressed as count (%). There were 85 missing values on the variable injury mechanism for patients undergoing isolated ACL-R

*ACL-R* isolated anterior cruciate ligament reconstruction, *BMI* body mass index, *ACL/PCL-R* combined anterior cruciate ligament and posterior cruciate ligament reconstruction, *PCL-R* isolated posterior cruciate ligament reconstruction

^a^Welch One-Way ANOVA with Games-Howell correction for multiple pairwise comparisons

^b^Chi-square test with a post hoc column pairwise comparison with Bonferroni correction

^c^Median (inter-quartile range)

^d^Kruskal–Wallis test with a post hoc column pairwise comparison using Bonferroni correction

There was a significant difference with respect to the mechanism of injury amongst groups, with a sports-related injury mechanism occurring predominantly in patients undergoing isolated ACL-R and a traffic-related injury mechanism in patients undergoing isolated PCL-R and combined ACL/PCL-R (*p* < 0.001). Concomitant meniscus injuries were reported in 45%, 31%, and 16% of patients with isolated ACL-R, combined ACL/PCL-R, and isolated PCL-R, respectively (*p* < 0.001).

### Functional outcomes

Detailed information on KOOS subscales for all groups collected preoperatively and at the 1- and 2-year follow-ups are presented in Tables 2, 3, and 4, respectively.

**Table 2:** Preoperative KOOS subscales

KOOS subscale	ACL-R (*n* = 30,577)	PCL-R (*n* = 81)	ACL/PCL-R (*n* = 73)	ACL-R vs. PCL-R (Diff., 95% CI)	ACL-R vs. ACL/PCL-R (Diff., 95% CI)	PCL-R vs. ACL/PCL-R (Diff., 95% CI)
Symptom	69 (19)	64 (18)	63 (17.7)	5 (1, 9)*	6 (2, 11)*	1 (− 5, 7)
Pain	74 (18)	65 (20)	64 (22.1)	9 (5, 13)**	10 (6, 14)**	1 (− 6, 8)
ADL	83 (18)	74 (21)	66 (22.9)	9 (5, 12)**	17 (13, 21)**	8 (2, 16)*
Sports/Rec	40 (28)	32 (26)	18 (19.9)	8 (2, 14)*	22 (16, 28)**	14 (6, 21)**
QoL	33 (19)	29 (17)	19 (18.0)	5 (1, 9)*	14 (10, 19)**	9 (4, 15)**

Continuous variables are expressed as mean (standard deviation). Comparison between groups was based on the Kruskal–Wallis test, followed by the Fisher’s non-parametric permutation test for pairwise group comparison

*ACL-R* isolated anterior cruciate ligament reconstruction, *ACL/PCL-R* combined anterior cruciate ligament and posterior cruciate ligament reconstruction, *ADL* activities of daily living, *Diff., 95% CI* mean and 95% confidence interval for the difference between groups, *PCL-R* isolated posterior cruciate ligament reconstruction, *QoL* knee-related quality of life, *Sports/Rec* sport and recreation function

*Statistically significant difference (*p* < 0.05)

**Statistically significant difference (*p* < 0.001)

**Table 3:** KOOS subscales at the 1-year follow-up

KOOS subscale	ACL-R (*n* = 23,656)	PCL-R (*n* = 85)	ACL/PCL-R (*n* = 99)	ACL-R vs. PCL-R (Diff., 95% CI)	ACL-R vs. ACL/PCL-R (Diff., 95% CI)	PCL-R vs. ACL/PCL-R (Diff., 95% CI)
Symptom	79 (18)	67 (15)	57 (20)	10 (4, 15)**	19 (12, 25)**	9 (0.6, 18)*
Pain	85 (16)	75 (16)	68 (19)	9 (4, 13)**	15 (9, 20)**	6 (− 1, 13)
ADL	92 (13)	85 (14)	75 (19)	5 (1, 9)*	14 (9, 18)**	9 (3, 15)**
Sports/Rec	65 (27)	43 (26)	32 (29)	20 (12, 29)**	30 (20, 39)**	9 (− 3, 22)
QoL	59 (23)	45 (22)	37 (22)	15 (7, 22)**	17 (8, 26)**	2 (− 9, 14)

Continuous variables are expressed as mean (standard deviation). Comparison between groups was based on a one-way ANCOVA controlling for preoperative subscale values, followed by post hoc pairwise comparison (Bonferroni method)

*ACL-R* isolated anterior cruciate ligament reconstruction, *ACL/PCL-R* combined anterior cruciate ligament and posterior cruciate ligament reconstruction, *ADL* activities of daily living, *ANCOVA* analysis of covariance, *Diff., 95% CI* mean and 95% confidence interval for the difference between groups, *PCL-R* isolated posterior cruciate ligament reconstruction, *QoL* knee-related quality of life, *Sports/Rec* sport and recreation function

*Statistically significant difference (*p* < 0.05)

**Statistically significant difference (*p* < 0.001)

**Table 4:** KOOS subscales at the 2-year follow-up

KOOS subscale	ACL-R (*n* = 18,854)	PCL-R (*n* = 72)	ACL/PCL-R (*n* = 85)	ACL-R vs. PCL-R (Diff., 95% CI)	ACL-R vs. ACL/PCL-R (Diff., 95% CI)	PCL-R vs. ACL/PCL-R (Diff., 95% CI)
Symptom	78 (18)	68 (18)	61 (20)	8 (1, 15)*	15 (8, 22)**	7 (− 3, 16)
Pain	85 (16)	74 (19)	70 (22)	7 (1, 13)*	10 (4, 16)**	3 (− 5, 12)
ADL	91 (14)	84 (18)	76 (21)	4 (1, 9)	9 (4, 14)**	5 (− 2, 12)
Sports/Rec	66 (27)	47 (30)	33 (30)	16 (6, 26)**	22 (11, 33)**	6 (− 9, 21)
QoL	61 (24)	51 (25)	40 (24)	8 (1, 17)	16 (6, 25)**	7 (− 6, 20)

Continuous variables are expressed as mean (standard deviation). Comparison between groups was based on a one-way ANCOVA controlling for preoperative subscale values, followed by post hoc pairwise comparison (Bonferroni method)

*ACL-R* isolated anterior cruciate ligament reconstruction, *ACL/PCL-R* combined anterior cruciate ligament and posterior cruciate ligament reconstruction, *ADL* activities of daily living, *ANCOVA* analysis of covariance, *Diff., 95% CI* mean and 95% confidence interval for the difference between groups, *PCL-R* isolated posterior cruciate ligament reconstruction, *QoL* knee-related quality of life, *Sports/Rec* sport and recreation function

*Statistically significant difference (*p* < 0.05)

**Statistically significant difference (*p* < 0.001)

#### Improvements in KOOS subscales over time

Significant improvements in all KOOS subscales were shown from the preoperative to 1- and 2-year follow-ups in patients undergoing isolated ACL-R (all *p* < 0.001). In patients undergoing isolated PCL-R, significant improvements in KOOS subscales (pain, activities of daily living, sport and recreation function, and knee-related quality of life) were shown from preoperative to 1 and 2 years postoperatively (all *p* < 0.05). Combined ACL/PCL-R resulted in significant improvements in KOOS subscales (activities of daily living, sport and recreation function, and knee-related quality of life) at 1- and 2-year follow-ups (all *p* < 0.05). However, patients undergoing combined ACL/PCL-R significantly deteriorated in the KOOS subscale symptoms (− 8.1 points) from preoperative to 1-year follow-up (*p* < 0.05).

**Fig. 2:**
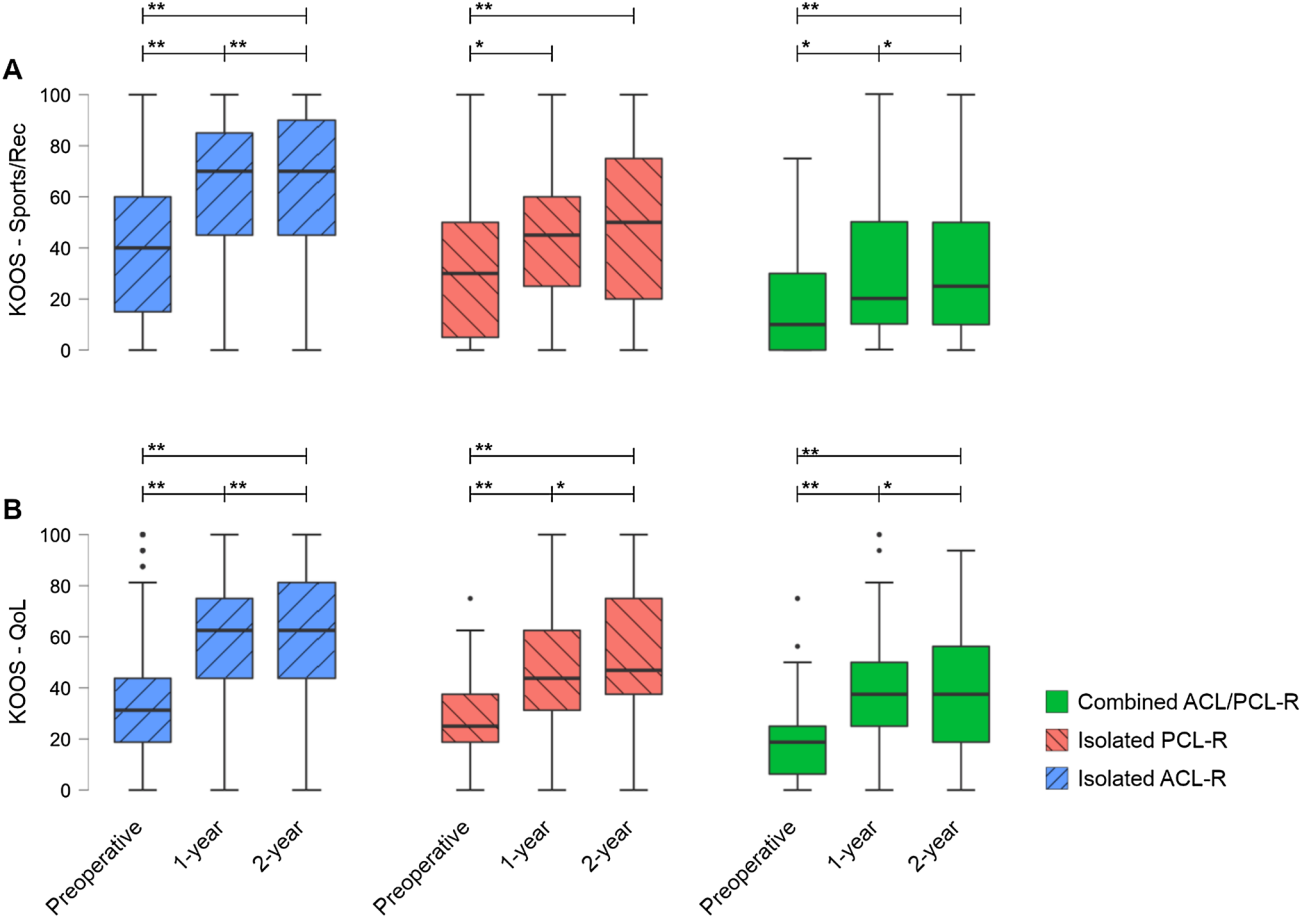
Improvements over time. KOOS (**A**) sport and recreation function (Sports/Rec) and (**B**) knee-related quality of life (QoL) subscales preoperatively and at the 1-year and 2-year follow-ups. Comparison over time was based on the Wilcoxon Signed Rank test. *ACL-R* anterior cruciate ligament reconstruction, *ACL/PCL-R* anterior cruciate ligament and posterior cruciate ligament reconstruction, *PCL-R* posterior cruciate ligament reconstruction; *Statistically significant difference (*p* < 0.05), **Statistically significant difference (*p* < 0.001)

All treatment groups showed the greatest improvements between the preoperative and 2-year follow-ups in the knee-related quality of life (mean improvement: isolated ACL-R, + 28 points; isolated PCL-R, + 23 points; combined ACL/PCL-R, + 21 points) and the function in sport and recreation (mean improvement: isolated ACL-R, + 26 points; isolated PCL-R, + 20 points; combined ACL/PCL-R, + 19 points) subscales (Fig. 2).

Patients undergoing isolated ACL-R improved significantly more in KOOS subscales sport and recreation function (25 vs. 10 points, *p* < 0.001) and knee-related quality of life (25 vs. 13 points, *p* < 0.001) than patients undergoing isolated PCL-R from preoperative to the 1-year follow-up. There was significantly more improvement in KOOS subscales symptoms (8 vs. − 8 points, *p* < 0.001), pain (10 vs. 1 points, *p* < 0.05), and sport and recreation function (25 vs. 10 points, *p* < 0.05) for patients undergoing isolated ACL-R compared to patients undergoing combined ACL/PCL-R from preoperative to the 1-year follow-up. In contrast, there was significantly more improvement in KOOS subscales symptoms (8 vs. 1 points, *p* < 0.001), pain (6 vs. 0 points, *p* < 0.001), activities of daily living (3 vs. 0 points, *p* < 0.05), and sport and recreation function (7 vs. 1 points, *p* < 0.05) for patients undergoing combined ACL/PCL-R compared to patients undergoing isolated ACL-R from 1- to 2-year follow-up. Patients undergoing combined ACL/PCL-R improved significantly more in KOOS subscales symptoms (8 vs. 1 points, *p* < 0.05) and pain (6 vs. 0 points, *p* < 0.05) from 1- to 2-year follow-up than patients undergoing isolated PCL-R.

## Discussion

The most important finding of this study was that regardless of the injury pattern of the central pivot (i.e. ACL, PCL), cruciate ligament reconstruction resulted in significant and clinically relevant improvements in knee function reflected by the KOOS. Functional improvements were most pronounced in the KOOS function in sport and recreation subscale, indicating the importance of knee stability for sports activity. Another pivotal finding was that patients undergoing isolated ACL-R showed higher preoperative and postoperative functional scores compared to injuries involving the PCL, reflecting the importance of the PCL for proper knee function.

Whilst ACL-R is recommended in young and athletic patients, there is controversy about the ideal treatment approach of isolated and combined PCL injuries [25, 27]. Concomitant meniscus injuries generally indicate surgical treatment to save as much meniscal tissue as possible in an attempt to protect the articular cartilage and prevent early-onset osteoarthritis. In this study, it was shown that patients undergoing isolated ACL-R (45%) were most likely to have concomitant meniscus injuries, followed by patients with combined ACL/PCL-R (31%), and isolated PCL-R (16%). The distribution of meniscus injuries is consistent with a previous study based on the NKLR displaying a prevalence of meniscus injuries of 49% and 11–12% in patients with ACL and PCL injuries, respectively [21]. The different proportions of meniscus injuries in ACL and PCL injuries may be attributable to different injury mechanisms. The majority of patients undergoing isolated ACL-R reported a sports-related injury mechanism (89%), commonly associated with a pivoting trauma, resulting in typically more stress on the menisci. In contrast, patients with isolated PCL-R (20%) and combined ACL/PCL-R (27%) had significantly higher proportions of traffic-related injuries. Notwithstanding a lower prevalence of concomitant meniscus lesions in injuries involving the PCL, preoperative knee function (involving all KOOS subscales) was significantly lower than for patients with isolated ACL-R. This finding corroborates with previous investigations from the NKLR and underscores the severity of injuries involving the PCL compared to isolated ACL injuries [2, 21].

Concomitant ligament injury has been shown to be a significant risk factor for graft failure after PCL-R [19]. However, no difference in functional outcomes between patients with isolated and combined PCL injury following PCL-R have been reported [10, 12, 20]. In one study, there was no difference between isolated (*n* = 77) and combined (*n* = 119) PCL-R in PROs (KOOS, IKDC-SKF, Tegner Activity Scale) and instrumented anterior–posterior laxity measurement (2.7 mm vs. 2.8 mm) after a mean follow-up of 5.9 years [18]. However, combined PCL-R included all types of PCL-based multiligament reconstructions. Accordingly, the difference between isolated PCL-R and combined ACL/PCL-R cannot be delineated. Whilst several case series have reported outcomes after combined ACL/PCL-R, comparisons to isolated ACL-R and isolated PCL-R are missing. In this study, KOOS subscales preoperatively and at 1 and 2 years of follow-ups were higher in patients with isolated PCL-R compared to patients with combined ACL/PCL-R. A previous investigation reported significant improvements in anterior–posterior knee laxity and PROs (IKDC-SKF, 90.6 points; Lysholm Score, 93.4 points) after 28 single-stage combined ACL/PCL-R using Achilles tendon allografts after a mean follow-up of 36 months [15]. A multicenter prospective study demonstrated similar results after combined ACL/PCL-R using hamstring tendon autografts for 20 patients after a mean follow-up of 26 months (IKDC-SKF, 90 points; Lysholm Score, 89 points, Tegner Activity Level, 7) [22].

Differences in postoperative outcomes after isolated ACL-R, isolated PCL-R, and combined ACL/PCL-R may be attributable to different patient-related and injury-related characteristics. Patients undergoing PCL-R are typically older and predominantly male compared to patients undergoing ACL-R [12, 13, 26]. Younger patients may be more ambitious and thus adhere more closely to the postoperative rehabilitation protocol, resulting in improved outcomes. In addition, patients undergoing PCL-R are often characterised by a significantly longer time from injury to surgery compared to patients with ACL-R [2, 12]. The extended preoperative time period may explain lower baseline scores and a higher prevalence of cartilage lesions in patients undergoing PCL-R compared to ACL-R [2, 21, 29].

Registry studies are subjected to several limitations including a large number of different surgeons with varying treatment philosophies for ACL, PCL, and combined ligament injuries, causing a certain heterogeneity in the investigated study cohorts. As PCL injuries are amenable to non-operative treatment, it would have been of high interest to include a non-operatively treated control group. Further limitations are the short follow-up period of only 2 years and the risk of unknown confounding factors which may affect the results. In addition, it was only possible to investigate differences in the KOOS, whereas other PROs would have been of high interest. However, the data used for this analyses derived from the SNKLR, covering more than 90% of all ACL-R in Sweden [1], making the results more generalizable. Based on patient expectations, the findings of this study may facilitate more individualised management in patients with isolated or combined ACL and PCL injuries.

## Conclusions

Clinically relevant improvements in knee function can be expected after isolated ACL-R, isolated PCL-R, and combined ACL/PCL-R. Isolated ACL-R results in superior knee function compared to isolated PCL-R or combined ACL/PCL-R. Injuries involving the PCL are severe; however, as demonstrated in this study, surgical treatment results in favourable patient outcomes. Functional improvements were particularly pronounced in the KOOS function in sport and recreation subscale, indicating the importance of knee stability for sports activity. The findings of this study facilitate more comprehensive patient education about functional expectations after surgical treatment of isolated and combined ACL and PCL injuries.
